# Circulating methylated promoters of HK2 and EGFR as biomarkers in the early detection of cancer

**DOI:** 10.3389/fonc.2026.1847436

**Published:** 2026-08-12

**Authors:** Mythreyi Jannu, Ramesh Ummanni, Rajeswari Jinka

**Affiliations:** 1 Department of Biochemistry, Acharya Nagarjuna University, Guntur, India; 2 Department of Applied Biology, CSIR-Indian Institute of Chemical Technology, Hyderabad, India

**Keywords:** circulating free DNA, DNA methylation, EGFR, HK2, promoter methylation

## Abstract

**Introduction:**

Aberrant methylation of promoter DNA regions is a well-known epigenetic hallmark of cancer. Previous studies on tumorigenic mouse models revealed that abnormal methylation in the promoter regions of selected genes occurs during the early stages of tumorigenesis. To explore further changes in the blood circulation of humans, we analysed the methylation levels of tumour- derived DNA in the plasma of selected samples, to develop a preliminary non-invasive method for early cancer detection.

**Materials and methods:**

Following earlier epigenetic modifications in tumorigenic mouse models, four genes (HK2, EGFR, DDIT3, and VEGFA) with differentially methylated promoters were selected for the present study. Targeted gene-specific methylated and unmethylated primers were designed by using the Methprimer program, and blood was drawn from clinically diagnosed cancer patients and healthy controls. cfDNA was isolated, bisulfite-treated, and analysed by qMSP. ROC curve analysis was conducted for genes that were found to be amplified, and validation was conducted by using *in silico* data from the MethMarkerDB across 10 cancer types.

**Results:**

Out of the four genes, HK2 and EGFR alone exhibited successful amplification and significantly higher methylation in the circulation of cancer samples. ROC analysis showed good diagnostic performance of HK2, AUC = 0.8288, and EGFR AUC = 0.9405. Relative methylation analysis in 12 different cancer types showed elevated methylation levels for HK2 and EGFR in liver, lung, prostate, ovarian, colon, stomach, breast, endometrial, and cervical cancers. *In silico* validation in the MethMarkerDB showed the existence of hypermethylation in tumour WGBS compared to healthy controls across 10 cancer types.

**Discussion:**

Abnormal promoter methylation is an early event in tumorigenesis and can be detected in circulating cfDNA. The present study analysed the diagnostic potential of HK2 and EGFR promoter methylation in cfDNA as non-invasive early cancer biomarkers. The results showed significant hypermethylation in cancer patients with strong diagnostic performance (AUC = 0.8288 and 0.9405).

## 1 Introduction

Cancer is a group of disease states characterised by the uncontrollable growth of cells that can invade and transform normal tissues. Its aetiology involves genetic and epigenetic alterations, as well as environmental factors, that promote its development, which eventually affect multiple body systems (1). Over 100 distinct types of cancers have been identified in humans. It is one of the most crucial health challenges of the 21st century, and the second-leading cause of death with a projected 20 million new cases and 9.7 million deaths in 2022 (2). In the United States, 2024 has been estimated to see 2,001,140 new cases and 611,720 deaths (3). Even with improvement in treatment, the prognosis is poor because most of the cancers were diagnosed at advanced stages. Early diagnosis is associated with improved survival rates, reduced treatment-related morbidity, and lower health expenditure. Nevertheless, the absence of symptoms in tumours occurring in early stages and inadequacies of existing diagnostic methods often prolong diagnosis. Therefore, developing sensitive, specific, and non-invasive diagnostic tests for cancer detection at an early stage is an essential clinical need (4, 5).

In recent years, liquid biopsy has emerged as a non-invasive diagnostic approach that involves the identification of tumour-derived components in body fluids like blood, urine, or saliva. It addresses the drawback of conventional tissue biopsies through real-time tumour profiling, illustrating the spatial and temporal heterogeneity, and facilitates repeated sampling during disease progression and treatment (6, 7). Increasing evidence enables its utility in early detection, prognostication, monitoring response to therapy, and assessment of minimal residual disease (8). Among all the liquid biopsy analytes, cell-free DNA (cfDNA) methylation patterns hold significant potential for early, non-invasive detection (9). Epigenetic changes, specifically aberrant deoxyribonucleic acid (DNA) methylation, are common, early, and reversible tumorigenesis events. DNA methylation involves the covalent attachment of a methyl group to the fifth carbon of cytosine bases in cytosine-phosphate-guanine (CpG) dinucleotides, which is a vital gene regulation event that does not change the sequence of DNA but regulates gene expression. It occurs mostly in CpG-rich sites known as CpG islands located in gene promoter regions and is crucial for gene expression control and genome stability (10, 11).

Aberrant DNA methylation is an early event in cancer, which involves focal hypermethylation of tumour suppressor genes and global hypomethylation. These changes can lead to chromosomal instability, aberrant gene expression, and oncogenesis (12). They are stable, replicable across sample types, and detectable in cfDNA in the circulation, and have benefits in biomarker development (13). Technological developments like quantitative methylation-specific PCR (qMSP) have facilitated more effective detection of methylated cfDNA. qMSP has high sensitivity, ease of use, and low cost, and is able to detect minute amounts of methylated alleles in the circulation. It has gained widespread application across various cancers for early diagnosis, monitoring, and recurrence surveillance (14).

Despite these advances, most of the clinically validated cfDNA methylation biomarkers are cancer-type specific. For example, the US FDA-approved septin 9 (SEPT9) methylation assay, commercially known as Epi proColon, is primarily used for colorectal cancer detection, whereas short stature homeobox 2 (SHOX2) and ras association domain family member 1A (RASSF1A)-methylation-based assays are used to detect lung cancer (15, 16). Several plasma-derived cfDNA methylation biomarkers have been investigated using methylation-specific PCR (MSP), quantitative MSP (qMSP), multiplex qMSP, and real-time MSP approaches across individual cancer types (17). For example, breast cancer biomarkers including estrogen receptor 1 (ESR1), SRY-box transcription factor 17 (SOX17), kallikrein-related peptidase 10 (KLK10), GATA-binding protein 3 (GATA3), and RASSF1A have shown potential as diagnostic and therapeutic monitoring markers (18). Similarly, lung cancer studies have evaluated adenomatous polyposis coli (APC), branched chain amino acid transaminase 1 (BCAT1), SHOX2, prostaglandin E receptor 4 (PTGER4), homeobox A9 (HOXA9), and KMT2C (lysine methyltransferase 2C) (19). In contrast, colorectal cancer studies have identified SEPT9, secreted frizzled related protein 2 (SFRP2), syndecan 2 (SDC2), branched chain amino acid transaminase 1 (BCAT1), IKAROS family zinc finger 1 (IKZF1), and EYA transcriptional coactivator and phosphatase 4 (EYA4) as promising methylation biomarkers (20). Likewise, genes such as HOXA9, hypermethylated in cancer 1 (HIC1), breast cancer 1 (BRCA1), and tissue factor pathway inhibitor 2 (TFPI2) have shown diagnostic relevance of cfDNA in ovarian cancer (21). These studies highlight the translational potential of plasma cfDNA methylation analysis and qMSP-based technologies for minimally invasive cancer detection. Despite these valuable findings, many studies have focused on evaluating gene methylation in specific cancer types for diagnosis, prognosis, or assessment of therapeutic response. Therefore, the applicability of these methylated biomarkers across multiple cancer types remains underexplored, particularly using minimally invasive plasma-derived cfDNA approaches (22). In addition, variability of diagnostic sensitivity across tumour stages and patient populations remains a challenge for cfDNA methylation biomarkers in clinical usage (23). Only a few studies have evaluated candidate gene methylation as multi-cancer biomarkers in large and diverse cohorts (24–26). Multi-cancer early detection (MCED) studies, including findings from the circulating cell-free genome atlas (CCGA), have demonstrated that methylation signatures are more effective than other genomic alterations for pan-cancer plasma-based detection (27). These MCED studies suggest the potential of detecting multiple malignancies using a single minimally invasive blood test, thereby improving cancer screening, detection, and clinical intervention. Furthermore, relatively few studies have evaluated plasma-derived cfDNA methylation biomarkers across multiple cancer cohorts using clinically applicable low-cost approaches such as qMSP (28–30). Therefore, there remains a significant need for robust, minimally invasive, and cost-effective cfDNA methylation biomarkers capable of detecting multiple cancers at early stages using clinically applicable approaches such as qMSP. Addressing this gap may facilitate the development of low-cost, minimally invasive multi-cancer screening strategies for cancer screening and early diagnosis.

In the present study, we aimed to identify clinically applicable cfDNA methylation biomarkers for early cancer detection using a minimally invasive and cost-effective qMSP-based approach. Unlike several recent MCED studies that primarily rely on genome-wide methylome sequencing, machine-learning algorithms, and computationally intensive bioinformatics workflows, these methods often require specialised infrastructure, advanced analytical expertise, and high implementation costs, thereby limiting routine clinical applicability (31, 32). Therefore, our study focused on the targeted evaluation of CpG island-rich promoter regions of selected candidate genes that are susceptible to aberrant methylation during tumorigenesis. Importantly, the present study evaluated plasma-derived cfDNA methylation using a cost-effective qMSP platform rather than large-scale sequencing-based approaches across a heterogeneous cohort comprising 12 cancer types, including a substantial proportion of Stage 0 (carcinoma *in situ* or equivalent, the earliest clinically diagnosed case) cases, to assess the potential utility of these biomarkers for early cancer detection. This targeted strategy provides a simpler, cost-effective, and minimally invasive approach for early-stage cancer detection across multiple cancer types.

In the current study, we analysed the methylation levels of four genes, namely hexokinase 2 (HK2), epidermal growth factor receptor (EGFR), DNA damage-inducible transcript 3 (DDIT3), and vascular endothelial growth factor A (VEGFA) in plasma-derived cfDNA between cancer patients and healthy controls. These genes evaluated in the present study were identified through a sequential experimental workflow. Initially, differential gene expression profiling was performed in a well-established tumourigenic mouse model (US Patent US9131668B2) developed using anoikis-resistant fibroblast-derived cells cultured under non-adherent conditions for 16 hours (F111 NA16 (cells), followed by malignant transformation, colony formation, and tumour development in nude mice (33). This model demonstrated several key hallmarks that closely resemble human tumorigenesis, including chromosomal instability, hypoxia-associated signalling, enhanced glycolytic activity, angiogenesis-related pathways, tumour formation, and cancer stem cell-like characteristics. Gene expression microarray analysis revealed alterations in genes that were involved in tumorigenesis, whereas the largest quantitative change in gene expression was found to be associated with pathways of hypoxia and glycolysis. These findings were further supported by quantitative real-time PCR, that identified dysregulation of 18 candidate genes including hypoxia inducible factor 1 subunit alpha (HIF1A), stanniocalcin 1 (STC1), VEGFA, von hippel-lindau tumor suppressor (VHL), DDIT3, Egl-9 family hypoxia inducible factor 3 (EGLN3), phosphofructokinase, muscle (PFKM), HK2, pyruvate dehydrogenase kinase 1 (PDK1), alcohol dehydrogenase 1 (ADH1), aldehyde dehydrogenase 3 family member A1 (ALDH3A1), solute carrier family 2 member 1 (SLC2A1), secreted phosphoprotein 1 (SPP1), matrix metallopeptidase 3 (MMP3), EGFR, and RB transcriptional corepressor 1 (RB1) (34). In a follow-up study of analysing circulating methylated biomarkers in transformed cell lines and tumourigenic nude mouse models, among the 18 genes, only seven genes (HIF1A, VEGFA, HK2, DDIT3, EGFR, SLC2A1, and PDK1) contained CpG islands in the promoter regions, these 7 genes were selected for methylation analysis using methylation-specific PCR (MSP). However, SLC2A1 and PDK1 did not show reproducible amplification and were excluded from further analysis. Although HIF1A showed partial methylation in transformed cells, it failed to demonstrate detectable amplification in the blood circulation of tumourigenic nude mice. In contrast, VEGFA, HK2, DDIT3, and EGFR demonstrated detectable abnormal changes in methylation status in transformed cells and circulatory blood samples of tumourigenic animals, indicating their potential detectability in circulation (35). Furthermore, extensive literature evidence has demonstrated the important roles of these genes in tumourigenesis, including glycolytic metabolic reprogramming of HK2 (36), oncogenic growth factor signalling of EGFR (37), cellular stress response and apoptosis regulation of DDIT3 (38), and increased angiogenesis of VEGFA (39). The tumourigenic nude mice were an initial model to identify genes associated with tumorigenic pathways, while the translational applicability of the selected genes was further supported through published human cancer studies (35–39). Therefore, these biological, experimental, and literature-based findings formed the basis for prioritizing HK2, EGFR, VEGFA, and DDIT3 for plasma cfDNA methylation analysis in human cancers. We used qMSP to detect the promoter methylation status of selected genes, which was able to distinguish cancer patients from controls in a minimally invasive plasma-based test. This strategy enables the development of cfDNA-based, methylation biomarkers for early cancer detection in various cancer types.

## 2 Materials and methods

### 2.1 Patients and clinical samples

This study included 200 histopathologically confirmed cancer patients and 100 healthy controls who were enrolled from the Department of Medical Oncology, Government General Hospital, Guntur. The cancer samples were collected in a non-selective manner without prior stratification based on cancer type. Following collection and subsequent clinicopathological evaluation, the cohort was categorized into 12 distinct cancer types, indicating a heterogeneous and generalized cancer population rather than a type-specific selection. Of the cancer cohort, 140 had stage 0 (carcinoma *in situ* or equivalent earliest stage), and the remaining samples had I-IV stage cancers of 12 different cancer types (**Table 1**). Healthy controls were verified with no history of malignancy. Informed consent was taken from all subjects, and ethical permission (GMC/IEC/27/2023) was obtained from the institutional ethics committee of Guntur Medical College. All experiments were performed following the institutional ethics committee and the Declaration of Helsinki. Demographic and clinicopathological information, age, sex, type of cancer, and stage were collected. Venous blood (5 ml) was drawn into BD Vacutainer^®^ K3 EDTA tubes and refrigerated at 4 °C. Plasma was separated through centrifugation at 1600×g for 10 min at 4 °C, preceded by a second centrifugation at 16000×g for 10 min (40). Plasma was aliquoted in 1.5 ml cryovials and refrigerated at **−**80 °C.

**Table 1:** Demographic characteristics of study participants, including cancer patients (n=200) and healthy controls (n=100).

Baseline characteristics	Cancer patients (n=200)	Healthy controls (n=100)
Age, years		
Mean	54.21	54.33
Sex n (%)		
Male	74 (37%)	46 (46%)
Female	126 (63%)	54 (54%)
Stage		
0 (carcinoma *in situ* or equivalent earliest stage)	140	–
I and II (combined)	20	–
III	14	–
IV	26	–

Data are presented as mean for continuous variables and as n (%) for categorical variables.

### 2.2 Extraction of DNA

cfDNA was isolated from 1 ml of frozen plasma by the HiPurA^®^ DNA extraction Kit (Himedia, MB504) according to the manufacturer’s instructions. DNA was eluted in 20 μl TE buffer and stored at −80 °C. Concentrations and purity were measured using a NanoDrop™ 2000/2000c Spectrophotometer (Thermo Scientific™) (41). The concentration of extracted cfDNA ranged from 1.8 to 2.0 ng/μL, with A260/A280 ratios between 1.6 and 1.9, indicating acceptable purity for downstream bisulfite conversion and qMSP analysis. Samples that failed quality requirements were replaced with adequate samples from the respective research group, and all analyses were performed on a final cohort of 200 cancer patients and 100 healthy controls.

### 2.3 Bisulfite conversion and purification

Bisulfite conversion of isolated DNA was performed according to the procedure described by Rajput et al. (2012) and Yi et al. (2017), with some adjustments. In a 0.2 ml PCR tube, 20 μl of DNA (~2 ng/μl) was denatured by adding 5 μl of 3 M NaOH at 50 °C for 15 min. Subsequently, 85 μl bisulfite solution and 25 μl tetrahydrofurfuryl alcohol (THFA) were added following the protocol of Rajput et al. (2012) (42). Thermal cycling conditions: 95 °C for 5 min, 55 °C for 25 min, 95 °C for 5 min, 55 °C for 85 min, 95 °C for 5 min, and 55 °C for 175 min. Desulfonation and purification were performed using appropriate buffers and a silica column-based method. DNA was eluted in 20 μl elution buffer (1 ng/μl) and stored at −20 °C. All the buffers required for bisulfite conversion and purification were freshly prepared as described by Rajput et al. (2012) and Yi et al. (2017) (42, 43), with minor modifications.

### 2.4 Primer designing

Primers for the targeted CpG-rich promoter regions of HK2, EGFR, VEGFA, and DDIT3 were designed using MethPrimer (http://www.urogene.org/methprimer/), a web-based program, by following a standardised sequential primer design workflow (44). This process involves, retrieval of transcription start site (TSS) positions using the database of transcriptional start sites (DBTSS) and promoter sequences from the eukaryotic promoter database (EPD), the evaluation of promoter region sequences between 1000 bp upstream and 500 bp downstream of the TSS for CpG dense domains suitable for methylation analysis and selection of optimal methylated and unmethylated primer pairs based on melting temperature (Tm) compatibility. Using this approach, primers for the promoter regions of HK2, EGFR, VEGFA, and DDIT3 were designed and synthesized by TechServ Healthcare Pvt Ltd. Chennai, India Detailed primer information is summarized in **Table 2**.

**Table 2:** qMSP primer details of HK2, EGFR, DDIT3, and VEGFA targeted promoter regions.

HUGO gene nomenclature	Chromosomal location	Amplicon annotation	CpG island length	No.of CpG islands	Forward primer sequence 5’-3’ (unmethylated)	Reverse primer sequence 5’-3’ (unmethylated)	Tm	Amplicon size	Forward primer sequence 5’-3’ (methylated)	Reverse primer sequence 5’3’ (methylated)	Tm	Amplicon size
HK2	Chr2(p12)	74834181- 74834380	200	1	AGAAAATTTGTGGGTTTGAGT	CAATTCTCTCCTAACAACATATCCAA	68.1	160	GTTAAGAAAATTCGCGGGTTC	CTCTCCTAACGACGTATCCGA	69.7	161
EGFR	Chr7 (p11.2)	55018778 -55018977	200	1	TTTTGTTGGAGATTAGGTTTTG	AACCAAACAACAAAAAAAAAATCA	66.8	144	TTCGTCGGAGATTAGGTTTC	AAACGACGAAAAAAAAATCGAA	66.4	149
DDIT3	Chr12(q13.3)	57520735 -57521034	300	1	GTTTACGATCGATTAGGGGC	ACGAAAATAATACAATATTTAACAACCG	65.8	173	ATGATTGATTAGGGGTGATT	ACAAAAATAATACAATATTTAACAACCAAT	67.8	169
VEGFA	Chr6(p21.1)	43769807-43770106	300	2	TTTTAGTAAAGAGGGAATGGTT	AATAAAACCTAACCCACAACCC	69.9	200	ATTTTTAGTAAAGAGGGAACGGT	CTCAATAAAACCTAACCCGCA	71.5	195

The table summarises chromosomal locations, CpG island details, primer sequences for both methylated and unmethylated alleles, Tm, and amplicon sizes.

### 2.5 Quantitative MSP

Following bisulfite conversion, the modified cfDNA was amplified using qMSP primers as per Herman et al. (1996) and Ogino et al. (2006) (45, 46) with minor modifications. Each 20 μl reaction comprised 2 µL bisulfite-converted DNA, 10 μl of 2X Genius SYBR Green Fast qPCR Master Mix (Cat. No: RK21204), 0.5 μl each of forward and reverse primers (10 μM), and 7 μl of nuclease-free water. Thermal cycling using a CFX Opus Real-Time PCR System (Bio-Rad) involved initial denaturation at 94 °C for 5 min; 40 cycles of 94 °C for 30 s, gene-specific annealing for 30 s, and 72 °C for 60 s; and final extension at 72 °C for 7 min. Primer sets and annealing temperatures are represented in **Table 2**. All runs had no-template controls and a reference gene, β2-microglobulin. PCR products were examined using 2% agarose gel electrophoresis and UV visualisation. Cycle Threshold (Ct) data were acquired with the single threshold protocol in Bio-Rad CFX Manager v3.1. Relative methylation (RM) was determined with the following formula: *Ct_M_* (methylated signal), *Ct_U_* (unmethylated signal), *Ct_ref_* (reference gene signal) (47).


$$\begin{array}{c} \Delta Ct_{M}=Ct_{M}-Ct_{ref}\\ \Delta Ct_{U}=Ct_{U}-Ct_{ref}\\ M=2^{-\Delta CtM}\\ U=2^{-\Delta CtU}\\ \text{Relative Methylation}=\frac{M}{M+U} \end{array}$$

### 2.6 *In silico* validation of targeted genes

To validate the selected genes as methylation biomarkers, we employed an *in silico* cancer methylation marker database known as MethMarkerDB (MetMarker database) (https://methmarkerdb.hzau.edu.cn/), a well-curated database containing more than 5.4 million differentially methylated regions (DMRs) for 658 whole-genome bisulfite sequencing (WGBS) datasets across 13 cancers with 724 biomarker genes from 1,425 PubMed articles (48). Using the cancer type drop-down menu and MethMarker region search, we retrieved methylation Beta-value (β-values) for the targeted promoter sequences of the selected genes for tumour and control samples across 10 cancer types. Beta-value represents the ratio of the methylated allele intensity to the overall intensity (sum of methylated and unmethylated allele intensities). Statistical comparisons of mean methylation β-values, standard errors, and p-values were performed in GraphPad Prism v9 (GraphPad Software, San Diego, CA, USA).

### 2.7 Statistical analyses

All the analyses and visualisations were performed with GraphPad Prism v9. A probability value (p-value<0.05) was used as statistically significant. Normality distribution of methylation data was performed using the Shapiro-Wilk test. As the data were not normally distributed, non-parametric Mann-Whitney U tests were performed to compare relative methylation levels between cancer and control groups. Multivariate logistic regression analysis was performed in Python using a Google Colab web application to assess the association between age, sex, HK2, and EGFR methylation with cancer status. We analysed the methylation levels of HK2 and EGFR across 12 different cancer types in clinical samples and 10 cancer types retrieved from the MethMarkerDB. The distribution and variability of relative methylation levels were assessed using dot plots between groups (age, gender, and cancer type) segregated based on demographic and clinicopathological data. To evaluate the diagnostic performance of HK2 and EGFR methylation, Receiver operating characteristic (ROC) curve analysis was performed, and the Area Under the Curve (AUC) values were calculated with 95% confidence intervals. The optimal cut-off value was determined using the maximum Youden index (sensitivity + specificity − 1) (49). A p-value of< 0.05 was considered statistically significant.

## 3 Results

### 3.1 Identification of CpG islands in target gene promoters

**Figure 1:**
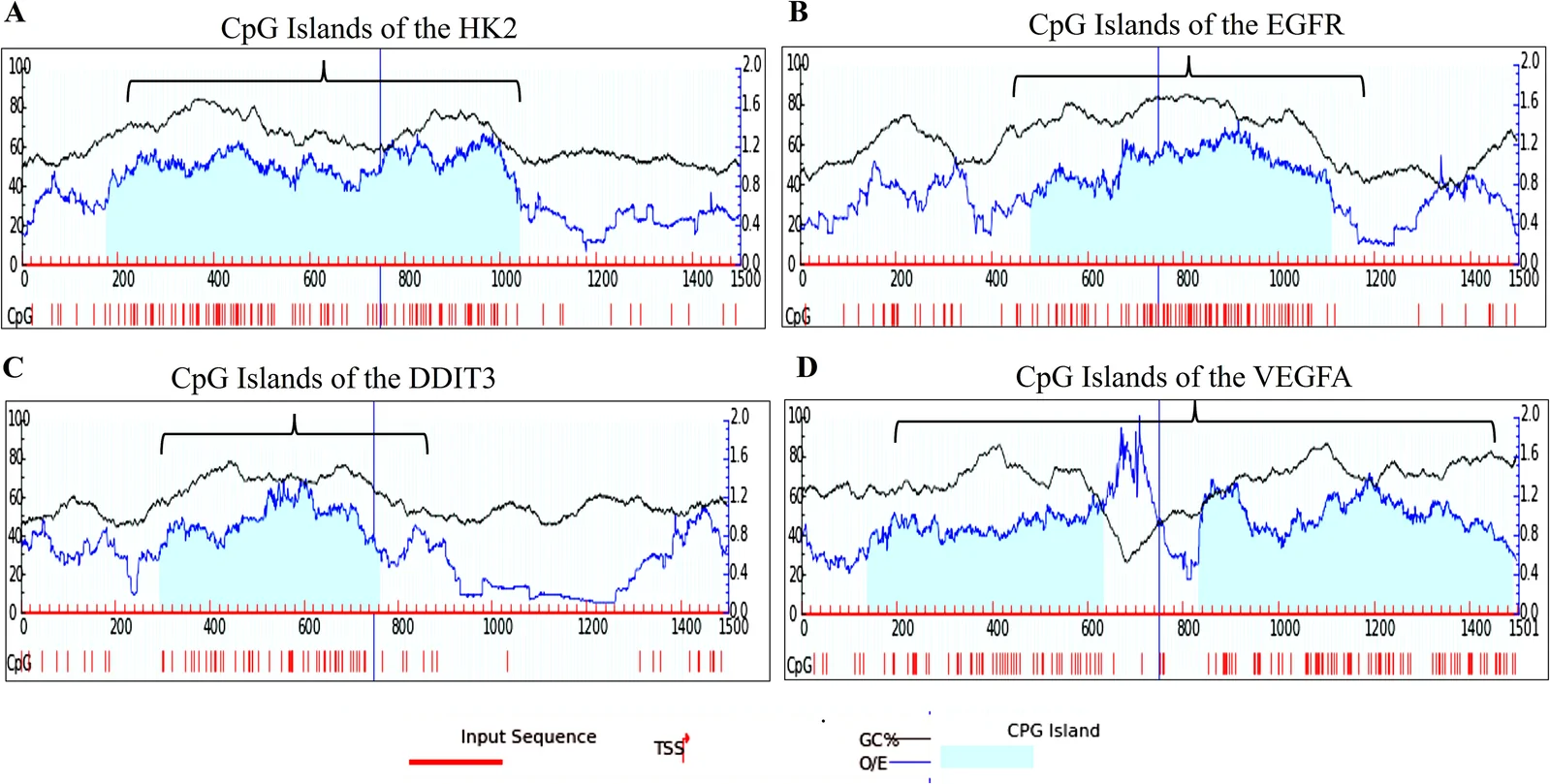
Identification of CpG islands in the promoter regions of **(A)** HK2, **(B)** EGFR, **(C)** DDIT3, and **(D)** VEGFA genes. Each panel displays the CpG island prediction plots for the respective gene using *in silico* analysis. The x-axis represents the nucleotide position across the input sequence, while the y-axis indicates the GC content (%) (black line, left axis) and the observed-to-expected CpG ratio (O/E) (blue line, right axis). CpG islands (light blue shaded regions) were defined based on regions with GC content >50%, O/E ratio >0.6, and length >200 bp. Vertical red bars at the bottom denote CpG dinucleotide positions across the sequence.

Using the MethPrimer program, CpG islands were identified within the promoter regions of the selected genes HK2, EGFR, DDIT3, and VEGFA. All genes exhibited CpG-rich sequences with GC (Guanine-cytosine) content above 50%, an observed/expected (O/E) ratio greater than 0.6, and lengths exceeding 200 bp (base pairs). Specifically, HK2 and EGFR each displayed one CpG island of 200 bp, DDIT3 displayed one CpG island of 300 bp, and VEGFA possessed two CpG islands of 300 bp each. Following identification of these CpG-rich promoter regions, methylated and unmethylated MSP primer sets were successfully designed and synthesised. Detailed CpG island illustrations and primer parameters, including methylated (M) and unmethylated (U) primer sequences, CpG island sizes, annealing temperatures, chromosomal coordinates, and amplicon sizes, are summarized in **Table 2**, **Figure 1**.

### 3.2 Detection of promoter methylation in cfDNA from cancer patients and healthy controls

**Figure 2:**
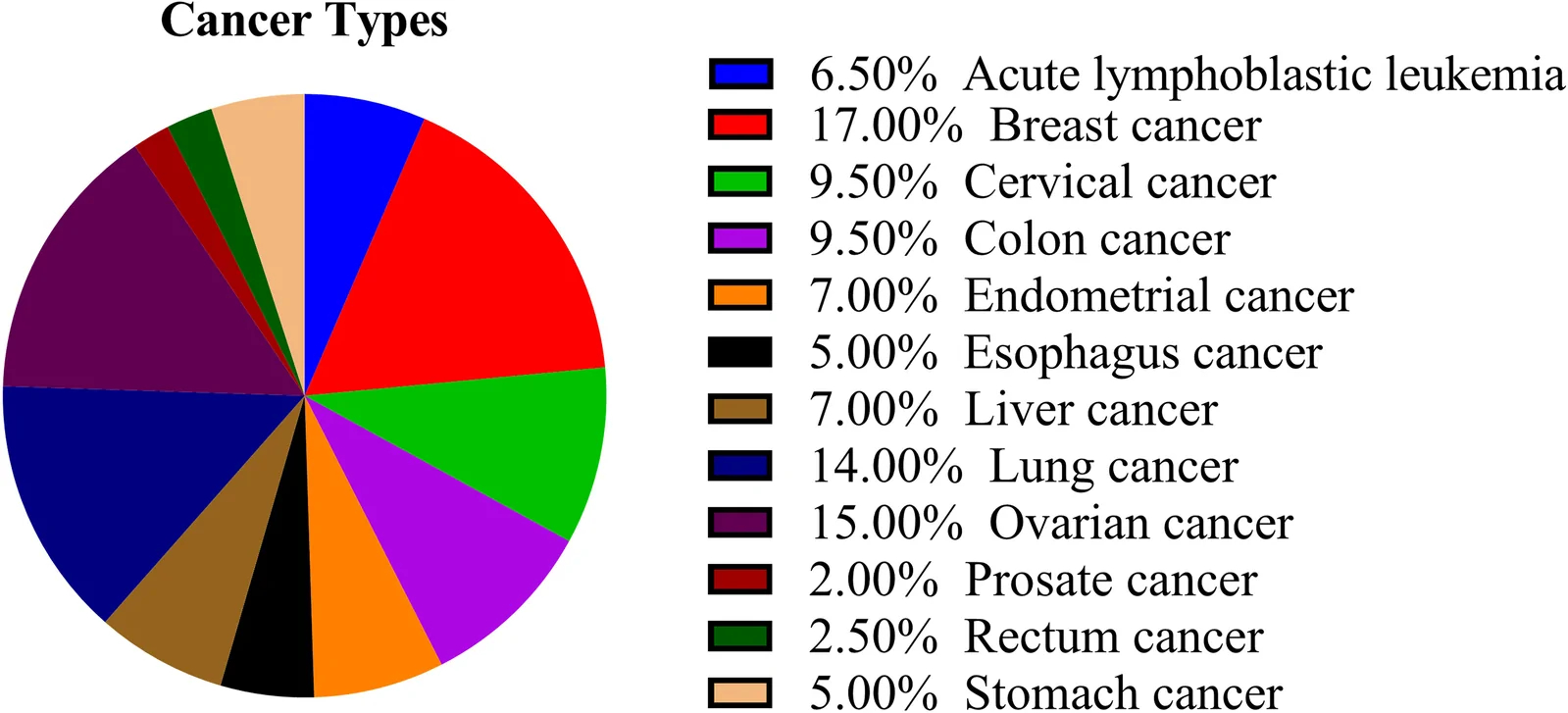
The pie chart represents the percentage distribution of 12 cancer types included in the cancer group (n=200).

**Figure 3:**
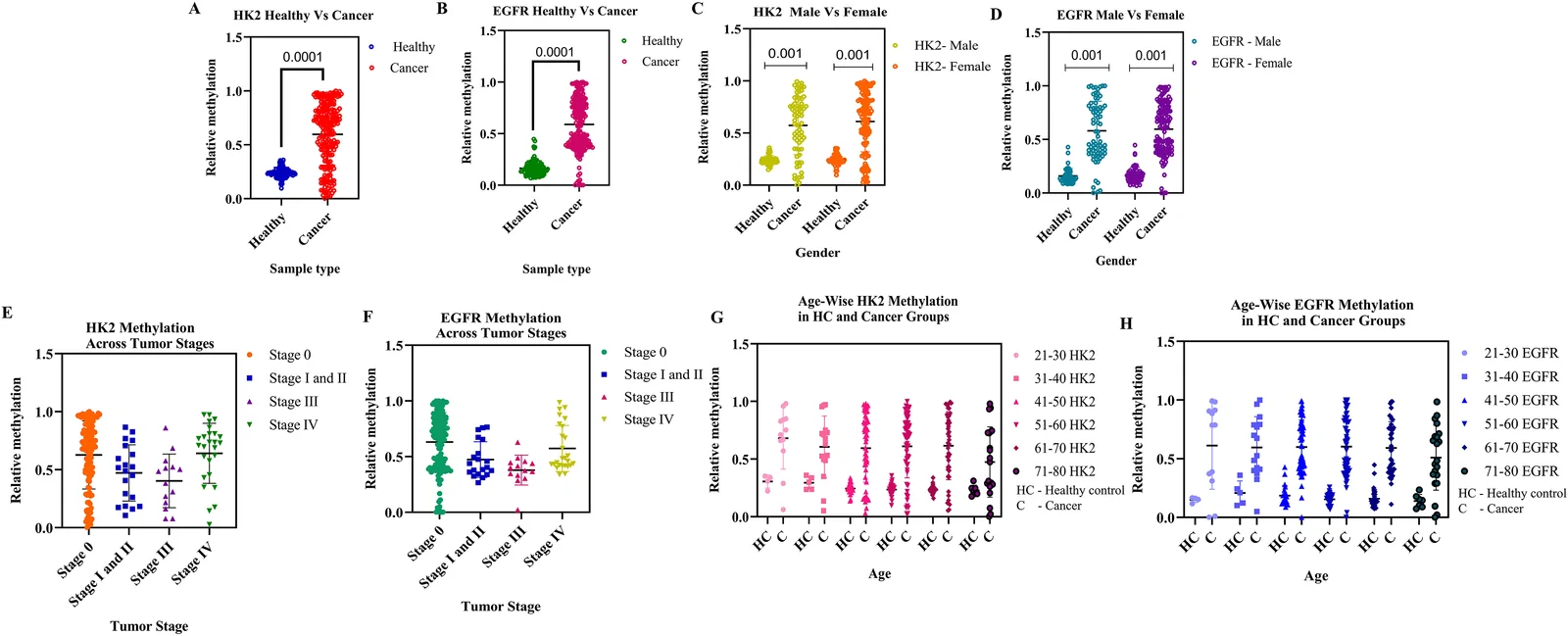
A dot plot illustrates the distribution of relative methylation values across various demographic characteristics, including sample group, gender, cancer stage, and age. Panels **(A, B)** show the comparison of relative methylation (RM) values of HK2 and EGFR between control and cancer groups, **(C, D)** demonstrate the RM values of HK2 and EGFR in male and female, **(E, F)** shows the RM values across cancer stages including stage 0, stage I–II (combined), stage III, and stage IV, with sample sizes per stage provided in **Table 1**. Panels **(G, H)** represent the comparison of RM values between cancer and control groups across different age groups, with sample sizes per age group provided in **Supplementary Table 1**. *p*-value statistical significance obtained by performing the Mann-Whitney U test (pairwise comparisons between healthy versus cancer, and male versus female groups).

A total of 200 blood samples from cancer patients across 12 cancer types and 100 age-matched healthy controls were included in the cfDNA methylation analysis (**Figure 2**). Extracted cfDNA demonstrated an average concentration of approximately 2 ng/μl, indicating sufficient yield and purity for downstream bisulfite conversion and qMSP-based methylation analysis. After bisulfite conversion, qMSP was performed to evaluate promoter methylation levels in HK2, EGFR, DDIT3, and VEGFA among 200 cancer samples and 100 healthy controls. The results revealed that among the four candidate genes, two genes, namely HK2 and EGFR, showed consistent amplification across the clinical samples, whereas DDIT3 and VEGFA failed to produce reliable amplification signals despite repeated optimization of PCR conditions; therefore, they were excluded from downstream analyses. The absence of detectable amplification may be associated with low abundance of methylated DDIT3 and VEGFA promoter regions within circulating cfDNA of the analyzed clinical cohort. Consequently, DDIT3 and VEGFA were excluded from downstream comparative and ROC analyses. To assess the usefulness of β2-microglobulin as a reference gene, we compared its *Ct* values between healthy controls (Median 22.22) and cancer samples (Median 22.25) using the Mann–Whitney U test. We observed that there was no significant difference between the two groups (p = 0.9153), supporting its applicability as an internal normalization control for qMSP analysis. Relative methylation analysis between cancer and healthy groups revealed that significantly elevated promoter methylation levels of HK2 and EGFR in cancer-derived cfDNA relative to healthy controls [**Table 3** (Clinical data); **Figures 3A, B**]. In the clinical cohort, the mean relative methylation level of HK2 increased from 0.2423 ± 0.0472 in healthy controls to 0.5971 ± 0.2871 in cancer samples (p< 0.0001, Mann–Whitney U test), while EGFR methylation increased from 0.1646 ± 0.0717 to 0.5897 ± 0.2528 (p< 0.0001). The observed elevation in promoter methylation of cancer-derived cfDNA suggests the presence of tumor-associated methylation dysregulation in selected gene promoters (50). These findings indicate that HK2 and EGFR methylation alterations were detectable in the circulating cfDNA of selected cancer patients.

**Table 3:** Comparison of methylation levels (mean ± SD) of selected genes between clinical data (comprising healthy and cancer samples) and data from the MethMarkerDB database (comprising healthy and cancer samples).

Gene	Clinical data			MethMarkerDB data		
	Healthy (mean ± SD) (n=100)	Cancer (mean ± SD) (n=200)	*P*-value	Healthy (mean ± SD) (n=338)	Cancer (mean ± SD) (n=281)	*P*-value
HK2	0.2423 ± 0.0472	0.5971 ± 0.2871	< 0.0001	0.1889 ± 0.1426	0.2648 ± 0.2296	0.0036
EGFR	0.1646 ± 0.0717	0.5897 ± 0.2528	< 0.0001	0.4758 ± 0.1957	0.6524 ± 0.2341	< 0.0001
DDIT3	—	—	—	0.7702 ± 0.1414	0.6726 ± 0.1891	< 0.0001
VEGFA	—	—	—	0.4377 ± 0.1415	0.4956 ± 0.1789	< 0.0001

Values are expressed as mean ± standard deviation (SD). *P-value* statistical significance was determined using the Mann-Whitney U test. p< 0.05 was considered statistically significant. “—” indicates data not available. Clinical data represent the experimentally obtained samples, while the *insilico* data are derived from the MethMarkerDB.

### 3.3 Analyses of promoter methylation in cancer vs. controls

Relative methylation analysis (2^-ΔCt) revealed significantly higher promoter methylation of HK2 and EGFR in cancer patients when compared to healthy individuals (p< 0.05), determined by the Mann-Whitney U test (**Figures 3A, B**). A similar trend was observed for both male and female groups with high relative methylation levels when compared to the controls (**Figures 3C, D**), indicating that comparable methylation patterns across sexes. Among the 200 cancer samples, 140 were at Stage 0, while the remaining samples were classified as stages I-IV. Stage-wise methylation analysis of HK2 revealed variable methylation levels from stage 0 to stage III, but persistently high methylation (>0.5) at stage IV (**Figure 3E**). EGFR also exhibited stage-independent methylation heterogeneity while maintaining overall higher methylation levels in cancer samples (**Figure 3F**), indicating that elevated HK2 and EGFR methylation is detectable among all stages of disease progression. Across all age groups (21–30, 31–40, 41–50, 51–60, 61–70, and 71–80 years), cancer samples showed significantly elevated methylation levels when compared to healthy controls for both genes (**Figures 3G, H**; **Supplementary Table 1**). Multivariate logistic regression results demonstrated that HK2 and EGFR promoter methylation remained independent predictors of cancer status after adjusting for age and sex. Age showed a significant association with cancer status odds ratio (OR) = 1.061, 95% confidence interval (CI): 1.007–1.118, p = 0.026), whereas sex did not show a significant effect (OR = 1.263, 95% CI: 0.378–4.223, p = 0.705) (**Supplementary Table 2**). Furthermore, these results indicate that circulating cfDNA hypermethylation of HK2 and EGFR is detectable across sex, age, and disease stage, although with inter-individual variability.

### 3.4 Performance of methylation markers across cancer types and ROC analyses

**Figure 4:**
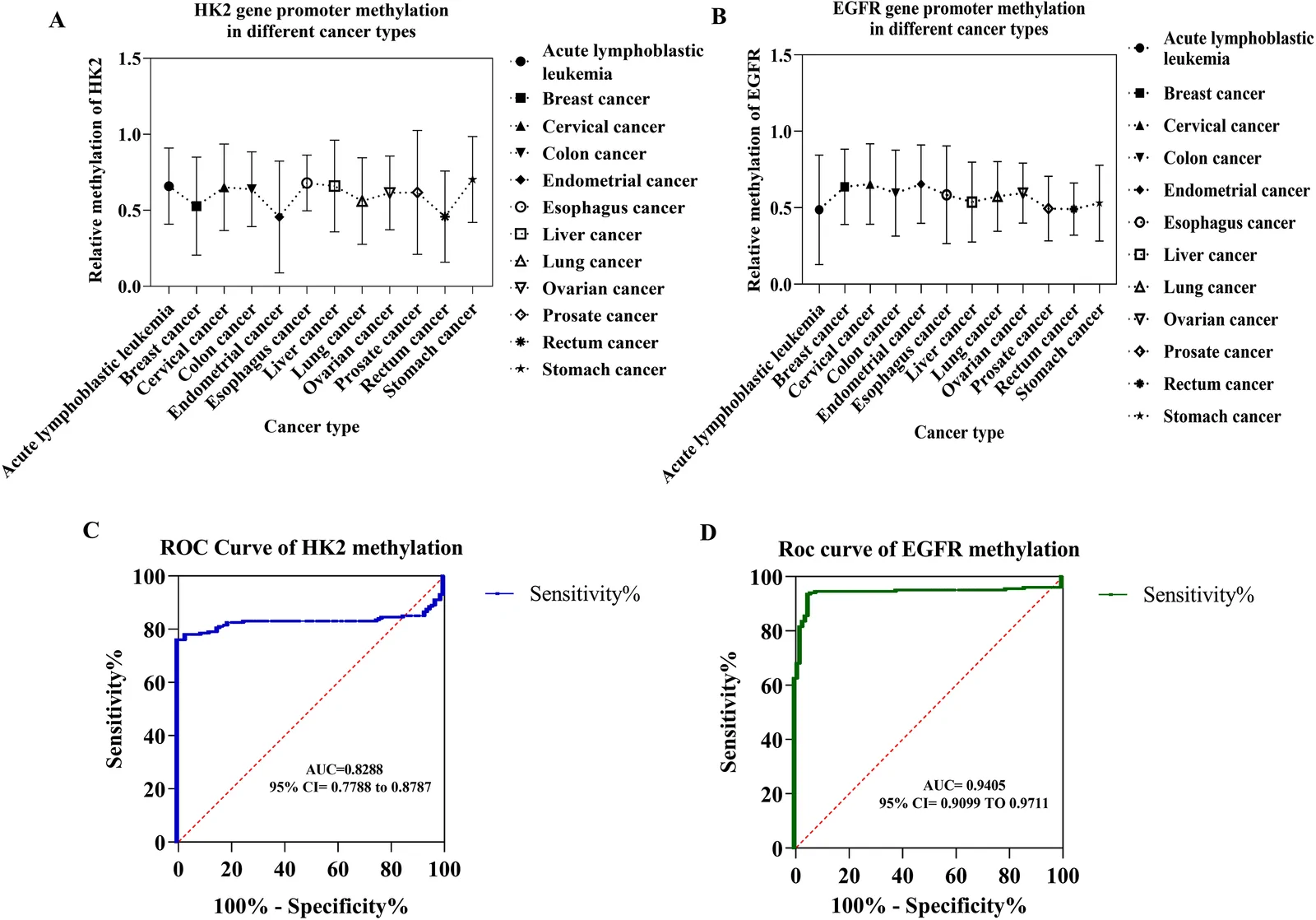
Cancer-type specific methylation distribution and diagnostic performance of HK2 and EGFR. Line graphs with error bars of panels **(A, B)** represent the relative methylation levels of the targeted promoter regions of HK2 and EGFR across 12 cancer types, respectively. **(C, D)** represent ROC curve analysis of HK2 and EGFR for distinguishing cancer patients from healthy controls. HK2 showed an AUC of 0.8288 (95% CI: 0.7788–0.8787), while EGFR demonstrated an AUC of 0.9405 (95% CI: 0.9099–0.9711). Each data symbol in **(A, B)** (circles, squares, triangles, diamonds) corresponds to a specific cancer subtype as indicated in the legend, and error bars represent the 95% confidence intervals (CI). The y-axis represents relative methylation values (0–1), and the x-axis represents the 12 malignancies included in the present study. **(C, D)** The red dashed diagonal line in the ROC curve represents the performance of a random classifier (AUC = 0.50).

We analysed the relative methylation values of HK2 and EGFR in 12 different types of cancer (**Supplementary Table 3**). The results of both genes revealed significant relative methylation values across different cancer types. Individual gene analysis revealed that HK2 was comparatively hypermethylated in cancers of the ovarian, cervical, colon, stomach, liver, prostate, and endometrium, whereas EGFR showed elevated methylation levels in cervical, ovarian, lung, breast, endometrial, and liver cancers. More variable cancers exhibited wider interquartile ranges, indicative of methylation heterogeneity (**Figures 4A, B**). ROC curve analysis demonstrated good diagnostic performance for HK2 and EGFR methylation in distinguishing cancer patients from healthy controls. HK2 showed an AUC of 0.8288 (95% CI: 0.7788-0.8787, p< 0.0001), with an optimized relative methylation cut-off threshold of 0.3091, yielding a sensitivity of 78.5% and a specificity of 90.0% (**Figure 4C**). EGFR exhibited a higher AUC of 0.9405 (95% CI: 0.9099-0.9711, p< 0.0001; **Figure 4D**). The optimal EGFR cut-off threshold of 0.2834, identified using the Youden Index, provided a sensitivity of 93.5% and a specificity of 95.0%.

#### 3.4.1 Specific diagnostic performance between stage 0 and healthy controls

**Figure 5:**
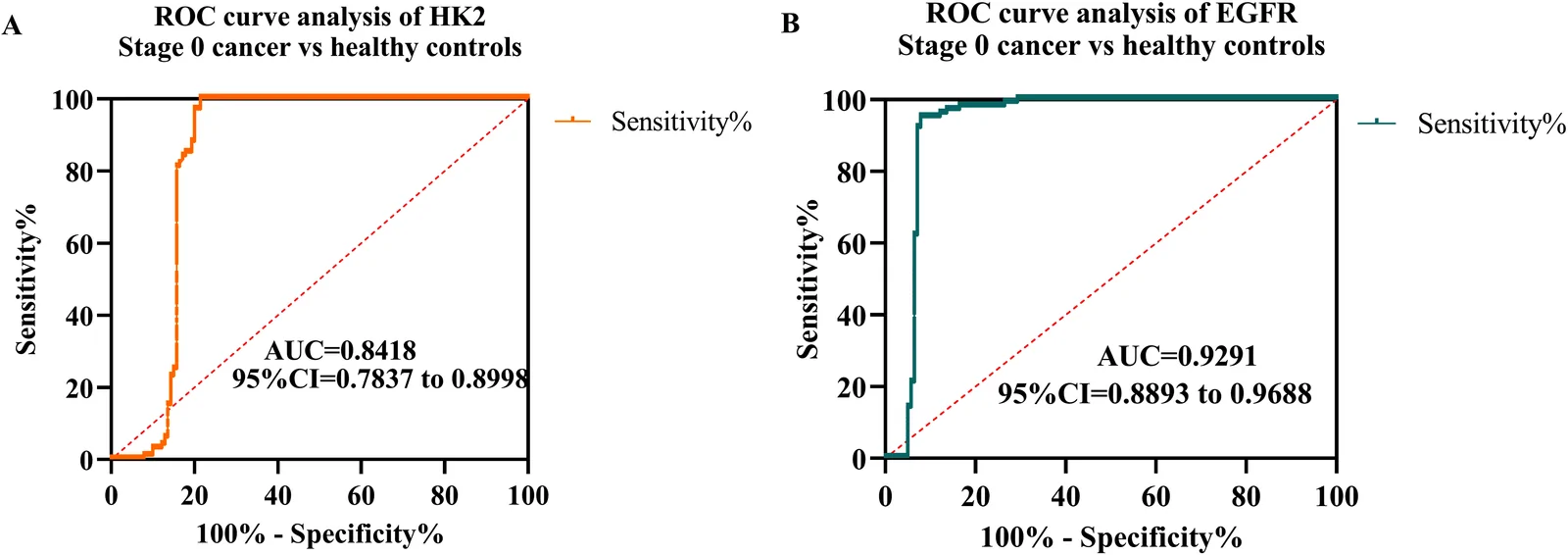
Evaluation of biomarker performance between stage 0 (n=140) and healthy controls (n=100). ROC curves demonstrate the ability of **(A)** HK2 promoter methylation and **(B)** EGFR promoter methylation to differentiate stage 0 cancer patients from healthy controls. EGFR methylation shows slightly higher diagnostic accuracy (AUC = 0.9291, 95% CI: 0.8893–0.9688) compared to HK2 methylation (AUC = 0.8418, 95% CI: 0.7837–0.8998). The red dashed line represents a random-chance classifier (AUC = 0.50).

We performed ROC analysis to examine the diagnostic efficacy of HK2 and EGFR in discriminating early cancer patients from healthy controls. Stage 0 (n = 140) cases were explicitly compared with healthy controls (n = 100). The results showed that HK2 demonstrated a discriminative ability with an AUC of 0.8418, producing a sensitivity of 97.0%, and a specificity of 80.0% at an optimal cut-off of 0.3404. Likewise, EGFR showed strong diagnostic performance with an AUC of 0.9292, sensitivity of 92.0%, and specificity of 92.86% at an optimal cut-off of 0.2510 (**Figures 5A, B**).

### 3.5 *In silico* validation using MethMarkerDB

**Figure 6:**
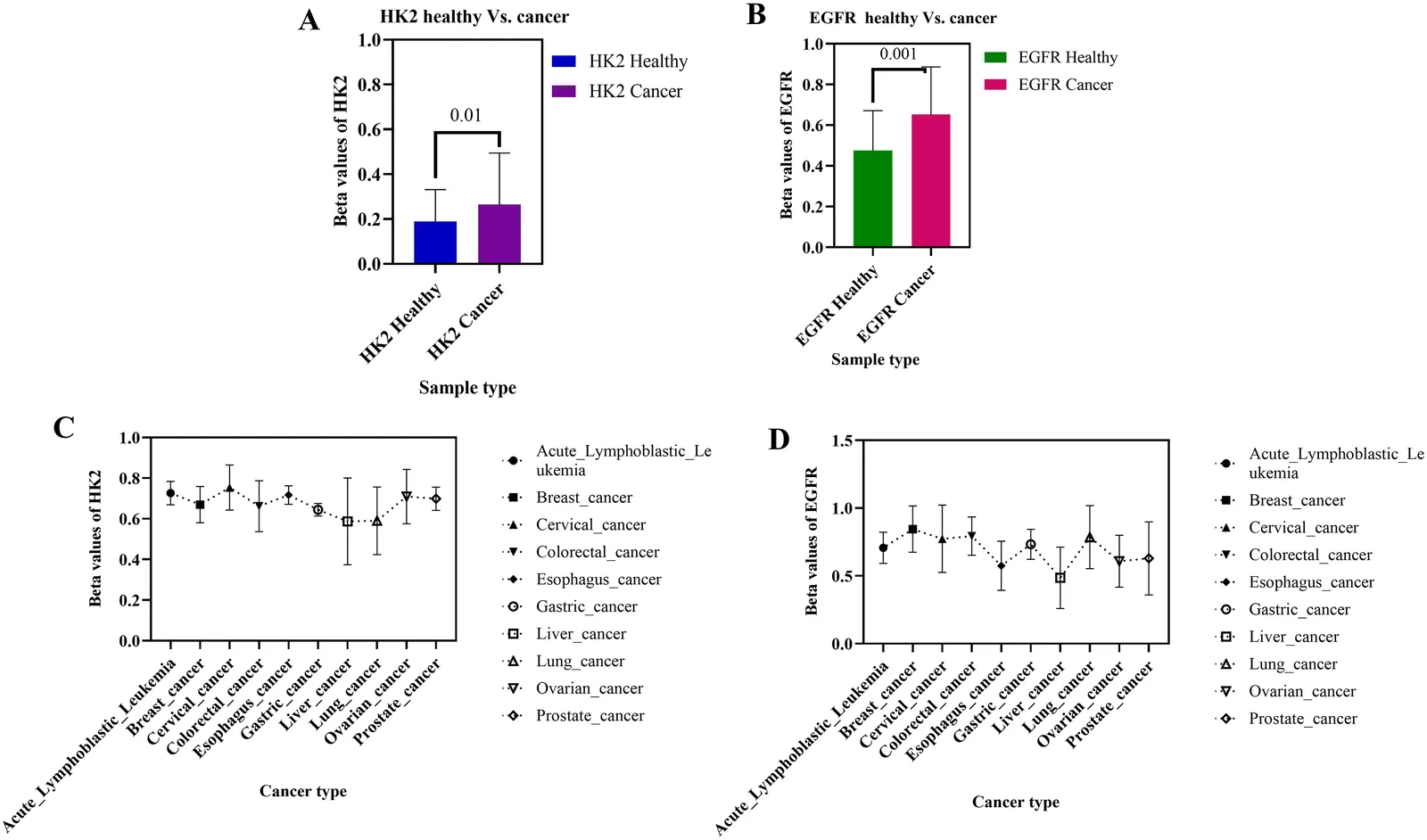
*In silico* validation of HK2 and EGFR β-values across healthy control (n=338) and cancer groups (n=281). **(A, B)** bar plots illustrating the DNA methylation β-values between healthy and cancer groups obtained from the MethMarkerDB. Panels **(C, D)** represent the line graphs with error bars showing the average DNA methylation β-values across different cancer types derived from the MethMarkerDB. Footnotes: In line graphs, each point represents the mean β-value, while the error bars indicate variability, reflecting differences in methylation levels among the cancer groups.

To validate the results further, we examined methylation patterns for HK2 and EGFR in the MethMarkerDB in 10 types of cancer (**Supplementary Table 4**). HK2 (located on chr2:74834127-74893359) and EGFR (located on chr7:55017969-55018403) promoter regions were highly hypermethylated in cancers compared to normal tissues (**Figures 6A, B**). In HK2, significant methylation increases were observed in cervical (p = 0.0168), ovarian (p< 0.0001), liver (p = 0.0243), lung (p = 0.0058), and prostate cancers (p = 0.0386). In EGFR, there were notable variations in breast (p< 0.0001), colorectal (p = 0.003), liver (p = 0.0088), lung (p< 0.0001), and prostate cancers (p< 0.0001), and also cervical and oesophageal tumours (**Figures 6C, D**). P-values were obtained by analysing MethMarkerDB data for each cancer type using the Mann–Whitney U test. ROC analysis of the MethMarkerDB data showed robust diagnostic potential. HK2 had very high AUCs for colon (0.903) (**Supplementary Figure 1A**), endometrial (0.962) (**Supplementary Figure 1B**), and lung (0.957) cancers (**Supplementary Figure 1C**). AUCs for EGFR were breast (0.953) (**Supplementary Figure 1D**), colon (0.900) (**Supplementary Figure 1E**), and prostate (0.954) (**Supplementary Figure 1F**) (Data obtained from MethMarkerDB). Our clinical ROC findings (AUC: HK2 = 0.8288, EGFR = 0.9405) were consistent with one another, supporting the diagnostic potential of these non-invasive cfDNA methylation biomarkers. We also analysed the methylations of promoter regions of DDIT3 and VEGFA from the MethMarker database. The results for DDIT3 indicated higher methylations in healthy individuals, whereas VEGFA indicated lower methylations in the targeted promoter region in the cancer group [**Table 3** (MethMarkerDB data)]. Studies on larger sample groups are required to validate the utility of methylated HK2 and EGFR in clinical applications.

## 4 Discussion

In this study, we analysed promoter methylation of HK2, EGFR, DDIT3, and VEGFA in plasma- derived cfDNA from cancer patients and controls using quantitative qMSP to evaluate their potential as non-invasive biomarkers in the detection of cancer at an early stage. Epigenetic modifications, particularly hypermethylation at the promoter region, are now established as early events in tumorigenesis (51) and have great potential as clinical diagnostics (52). As these modifications tend to occur early during the multistep process of tumorigenesis (53), the identification of methylation in cfDNA can provide insight into incipient cancer formation even before the detection of clinically evident tumours. Therefore, it might be insufficient to just depend on one methylation marker because tumorigenesis is a multistep process that involves various other factors as well (54). Hence, there is a growing interest in identifying methylation biomarkers capable of detecting cancer at earlier stages across diverse cancer types rather than individual tumour-specific markers (55). The present study focused on evaluating promoter methylation of selected genes HK2, EGFR, DDIT3, and VEGFA in plasma-derived DNA of 12 different cancer types using qMSP. qMSP is extremely sensitive, economical, and can detect low quantities of tumour DNA in blood with significant advantages over conventional diagnostic tests (56). Our qMSP analysis showed significantly higher promoter methylation of HK2 and EGFR in cancer patients, implying their diagnostic utility. Despite multiple optimisation attempts, including adjustments in primer concentration, annealing temperature, and cycle number, no specific amplification was observed for DDIT3 and VEGFA, possibly due to the lower abundance of these methylated promoter regions in circulating human cfDNA or greater biological heterogeneity across cancer types (57). Interestingly, MethMarkerDB analysis also showed no significant methylation changes of the targeted promoter regions of DDIT3 and VEGFA between cancer and control groups, providing additional support for their exclusion from downstream analyses. Methylation profiling of DNA across multiple frequently aberrant loci enhances the diagnostic sensitivity for non-invasive detection (58). A recent study demonstrated the bisulfite-free qMSP-like approach reliably detected TAG of methylation (TAGMe) in urine from multiple cancers, including urothelial carcinoma, and identified recurrence before clinical detection (4). Another study emphasised that the utility of qMSP in plasma-based short stature homeobox 2 (SHOX2) and ras association domain family member 1, isoform A (RASSF1A) methylation detection was promising in early-stage lung adenocarcinoma, with AUC values of 0.806, competitive with invasive procedures such as bronchoalveolar lavage. This approach offers a less invasive yet effective alternative for lung cancer detection. The study’s generalizability was constrained by a small early-stage lung adenocarcinoma (LUAD) cohort, the absence of healthy controls, and technical limitations in cfDNA yield from plasma (59). In prostate cancer, cysteine dioxygenase type 1 (CDO1) methylation detected by qMSP correlated with decreased gene expression and could serve as a potential prognostic biomarker for biochemical recurrence (BCR)-free survival following radical prostatectomy, highlighting the value of qMSP as a reliable technique for methylation-based prognostic biomarker detection in cancer. The prognostic utility of CDO1 methylation was limited by its failure to retain significance in multivariate analysis and by the short follow-up duration in The Cancer Genome Atlas (TCGA) data (60). These findings highlight the potential of qMSP-based methylation assays for non-invasive, multi-cancer diagnosis. Unlike many recently reported multi-cancer early detection studies that rely on genome-wide methylation sequencing and machine-learning algorithms (27–32), the present study implemented a targeted qMSP-based technique, which is a more cost-effective and clinically accessible alternative for preliminary multi-cancer screening applications.

Large-scale methylome analysis with TCGA data has further proven the diagnostic potential of DNA methylation. Vrba and Futscher (2018) (26) have identified high-performing pan-cancer and cancer-specific CpG panels, including well-known markers like SEPT9 and glutathione S-transferase Pi 1 (GSTP1) and novel candidates such as MIR129-2, with good diagnostic performance (AUC: 0.969-1.000) by using TCGA, whereas Ibrahim et al. (2022) (61) have also reported 1,991 pan-cancer and numerous type-specific differentially methylated CpGs by performing comprehensive methylome analysis across 14 cancer types. Their optimised four-marker panel achieved an AUC of 0.90 signifies the importance of methylation profiling in non-invasive detection. Previously, Ibrahim et al. (2019) (27) conducted a TCGA-based study on Gasdermin E, and the results showed high diagnostic accuracy (AUC = 0.86–0.97) for both TCGA and external validation datasets. Our observations were aligned with these investigations. The diagnostic performance observed in the present study was supported by ROC analysis, which demonstrated good discriminatory power for HK2 (AUC = 0.8288) and excellent performance for EGFR (AUC = 0.9405), indicating that promoter methylation of these genes can effectively distinguish cancer patients from healthy individuals. The consistency between clinical cfDNA methylation findings and the methylation data obtained from MethMarkerDB further strengthens the biological relevance of HK2 and EGFR. The diagnostic accuracy of both markers to distinguish stage 0 from healthy controls supports their potential as early cancer detection biomarkers. Notably, elevated methylation levels were consistently observed across age groups, sexes, and disease stages, supporting the broad detectability of HK2 and EGFR methylation in the analysed cohort. Multivariate analysis further indicated that these correlations were independent of age and sex, suggesting their clinical potential. Although the findings are encouraging, the present study has limitations. First, this study provides an initial assessment of the selected cfDNA methylation markers; however, an independent external validation cohort was not available during the study period. Future large-scale, independent study cohorts are necessary to establish the robustness and generalizability of our findings. In addition to this, our *in silico* validation was based on tissue-derived whole-genome bisulfite sequencing data retrieved from MethMarkerDB, whereas the present study evaluated plasma-derived cfDNA. Because methylation patterns can change between tissue and plasma cfDNA, differences between the clinical and *in silico* results should be interpreted in the context of this limitation. Second, cfDNA quality assessment was based primarily on concentration and purity, characterization of the cfDNA fragment-size analysis was not performed. Despite these limitations, the present findings provide evidence supporting HK2 and EGFR promoter methylation as promising cfDNA-based biomarkers for minimally invasive early-cancer detection in selected multi-cancer cohorts.

## 5 Conclusion

This study evaluated promoter methylation alterations in four candidate genes, which were selected based on the previous tumorigenic mouse model data. By using gene-specific qMSP primers, we examined bisulfite-converted cfDNA from cancer patient and control subject plasma using qMSP. Two of the four genes, HK2 and EGFR, showed significantly higher methylation levels in the cancer samples, whereas VEGFA and DDIT3 did not show amplification and were not included in further analysis. ROC analysis demonstrated high diagnostic promise, particularly for EGFR (AUC = 0.9405). Additionally, *in silico* validation with the MethMarkerDB from 10 different cancers also validated consistent HK2 and EGFR hypermethylation across multiple cancer types. These results collectively suggest that HK2 and EGFR are noninvasive, promising methylated biomarkers for cancer detection in the early stages.

## Data Availability

The raw data supporting the conclusions of this article will be made available by the authors, without undue reservation.
