# Supplementary material for: Circulating methylated promoters of HK2 and EGFR as biomarkers in the early detection of cancer
Source: Front Oncol. 2026 Aug 12;16:1847436. doi: 10.3389/fonc.2026.1847436 (PMC13506309; doi:10.3389/fonc.2026.1847436)
Supplement: Supplementary file 1 [file SupplementaryFile1.docx]

Supplementary Material

# 1 Supplementary Table S1 Distribution of samples by age group in the selected cohort of 200 cancer patients and 100 healthy controls.

| **Age Group** | **Cancer patients (n)** | **Healthy controls (n)** |
| --- | --- | --- |
| 21–30 | 12 | 4 |
| 31–40 | 16 | 5 |
| 41–50 | 51 | 22 |
| 51–60 | 58 | 28 |
| 61–70 | 42 | 34 |
| 71–80 | 21 | 7 |
| Total | 200 | 100 |

# 2 Supplementary Table S2 Multivariate logistic regression analysis assessing the association of age, sex, HK2 methylation, and EGFR methylation with cancer status.

| **Variable** | **β** | **SE** | **OR (95% CI)** | **p-value** |
| --- | --- | --- | --- | --- |
| Age | 0.059 | 0.027 | 1.061 (1.007–1.118) | 0.026 |
| Sex (male vs female) | 0.233 | 0.616 | 1.263 (0.378–4.223) | 0.705 |
| HK2 methylation | 14.154 | 2.559 | 1.40 × 10⁶ (9.3 × 10³–2.1 × 10⁸) | <0.001 |
| EGFR methylation | 19.055 | 3.079 | 1.89 × 10⁸ (4.5 × 10⁵–7.9 × 10¹⁰) | <0.001 |

Footnotes: β - regression coefficient, SE - standard error; OR - odds ratio, CI - confidence interval. Odds ratios were estimated from a multivariable logistic regression model including age, sex, HK2 methylation, and EGFR methylation.

# 3 Supplementary Table S3. Distribution of 12 cancer types in the cohort of 200 cancer patients.

| **Cancer Type** | **No.of samples (n)** |  |
| --- | --- | --- |
| Acute lymphoblastic leukemia | 13 |  |
| Breast cancer | 34 |  |
| Cervical cancer | 19 |  |
| Colon cancer | 19 |  |
| Endometrial cancer | 14 |  |
| Esophagus cancer | 10 |  |
| Liver cancer | 14 |  |
| Lung cancer | 28 |  |
| Ovarian cancer | 30 |  |
| Prostate cancer | 4 |  |
| Rectum cancer | 5 |  |
| Stomach cancer | 10 |  |
| Total | 200 |  |

**Supplementary Table S4.** *In silico* validation across 10 cancer types, data retrieved from MethMarkerDB, representing an independent public dataset analysis.

| **Cancer Type** | **No.of samples in Healthy group (n)** | **No.of samples in Cancer group (n)** |
| --- | --- | --- |
| Acute lymphoblastic leukemia | 117 | 18 |
| Breast cancer | 10 | 24 |
| Cervical cancer | 10 | 13 |
| Colorectal cancer | 18 | 30 |
| Esophagus cancer | 51 | 52 |
| Gastric cancer | 3 | 3 |
| Liver cancer | 50 | 53 |
| Lung cancer | 31 | 29 |
| Ovarian cancer | 34 | 28 |
| Prostate cancer | 14 | 31 |
| Total | 338 | 281 |

**Supplementary Figure 7** Diagnostic performance of HK2 and EGFR methylation in specific cancer types. Panels **(A)**, **(B)**, and **(C)** represent the ROC curves evaluating the diagnostic performance of HK2 in Endometrial cancer, Colon cancer, and Lung cancer, respectively. **(D)**, **(E)** and **(F)** illustrate the ROC curves of EGFR in Breast cancer, Colon cancer, and Prostate cancer, respectively. Footnotes: The solid orange lines represent the ROC curves for each biomarker, while the diagonal dashed black lines serve as the reference for a random classifier (AUC = 0.5). Each panel includes the AUC values followed by their corresponding 95% confidence intervals in parentheses (Data retrieved from MethMarkerDB).
